# Type IIB PKA serves as the primary effector of Gs-coupled receptor-potentiated insulin secretion in mice by orchestrating ion channels and granule phenotype

**DOI:** 10.1007/s00125-026-06781-8

**Published:** 2026-06-23

**Authors:** Ying Liu, Yunzhi Ni, Chuantong Xie, Linling Fan, Yaojing Jiang, Quanya Sun, Bei Mao, Chuxin Huang, Zhaoyun Zhang, Yehong Yang, Min He, Lei Xiao, Yiming Li, Rui Liu

**Affiliations:** 1 Department of Endocrinology and Metabolism of Huashan Hospital, State Key Laboratory of Brain Function and Disorders, MOE Frontiers Center for Brain Science and the Institutes of Brain Science, Fudan University, Shanghai, China; 2 Institute of Endocrinology and Diabetes, Fudan University, Shanghai, China; 3 Shanghai Clinical Research and Trial Center, ShanghaiTech University, Shanghai, China

**Keywords:** Glucagon-like peptide-1, G-protein-coupled receptor, Insulin secretion, Protein kinase A

## Abstract

**Aims/hypothesis:**

G-protein-coupled receptors (GPCRs) play an important role in maintaining systemic glucose homeostasis by regulating insulin secretion, with protein kinase A (PKA) signalling serving as a key downstream effector. Our previous work identified specific expression of type IIB PKA in pancreatic beta cells. Based on these findings, we propose that type IIB PKA is involved in mediating the GPCR signalling in pancreatic beta cells.

**Methods:**

The glucagon-like peptide-1 (GLP-1) analogue liraglutide was administered to mice 30 min before glucose injection during an IPGTT, whereas the glucose levels and insulin levels were measured in wild-type and RIIβ-knockout mice. The isolated islets were subjected to both perifusion assay and static batch incubations following stimulation with liraglutide, glucagon and follicle-stimulating hormone (FSH). RNA-seq analysis was performed to identify molecular changes in islets with RIIβ ablation. Both western blotting and quantitative PCR were employed to quantify the gene expression. Whole-cell patch-clamp recordings were conducted to measure K_ATP_ and Ca^2+^ currents. Insulin granule morphology and abundance were evaluated by electron microscopy and flow cytometry using EGFP-labelled Syncollin, respectively.

**Results:**

RIIβ-knockout mice exhibited impaired glucose tolerance and attenuated insulin secretion in response to liraglutide. Islets isolated from RIIβ-knockout mice showed reduced insulin secretion following liraglutide stimulation. Similarly, RIIβ-ablated islets displayed decreased insulin secretion in response to both glucagon and FSH. Further mechanistic studies revealed that RIIβ deficiency impaired liraglutide-mediated PKA signalling activation. Specifically, RIIβ-ablated beta cells exhibited reduced basal K_ATP_ channel activity and lack of liraglutide-mediated channel inhibition. Multiple voltage-gated Ca^2+^ channel genes were downregulated in RIIβ-ablated islets, leading to a mild reduction in basal Ca^2+^ current and a significant decrease following liraglutide treatment. RIIβ-knockout beta cells also exhibited reduced insulin granule size, decreased total granule number and fewer granules docked at the plasma membrane.

**Conclusions/interpretation:**

Our results highlight type IIB PKA as a primary mediator of Gs-coupled receptor-potentiated insulin secretion, providing a new molecular framework for metabolic regulation research.

**Graphical Abstract:**

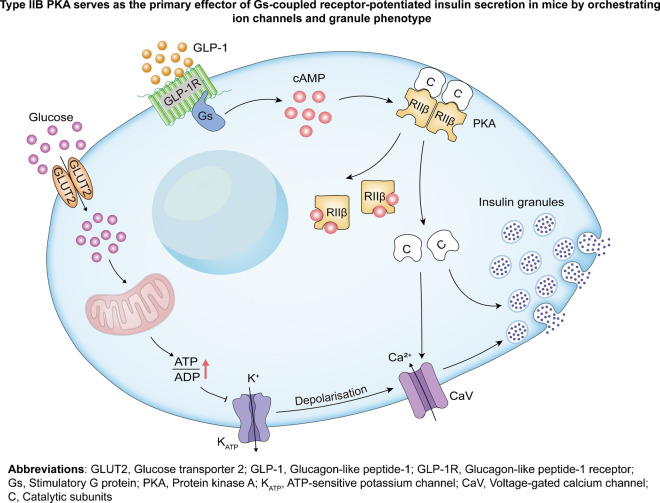

**Supplementary Information:**

The online version contains peer-reviewed but unedited supplementary material available at 10.1007/s00125-026-06781-8.


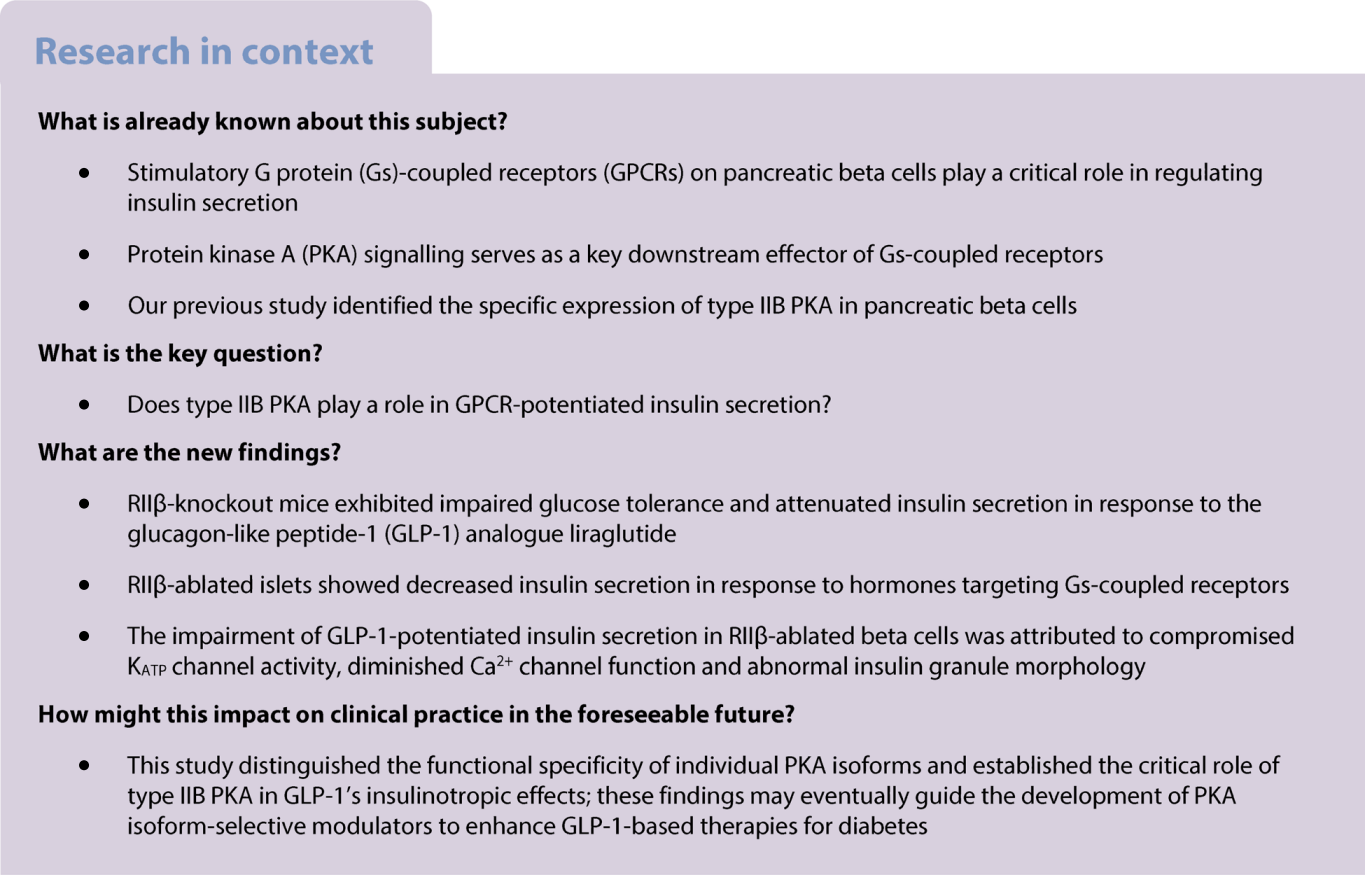


## Introduction

The pancreatic islets mainly harbour two different endocrine cell populations distinguished by their unique hormone products: insulin-producing beta cells; and glucagon-secreting alpha cells. These cells collectively maintain the blood glucose level under precise control through their complementary hormonal actions [1]. Of particular importance, insulin is the key hormone responsible for postprandial glucose disposal by promoting cellular glucose uptake in peripheral tissues. When beta cell dysfunction leads to insufficient insulin secretion, glycaemic regulation is compromised, resulting in elevated blood glucose levels and the onset of diabetes [2].

Insulin secretion from beta cells is potently activated by a postprandial increase in circulating glucose concentrations, referred to as glucose-stimulated insulin secretion (GSIS) [3, 4]. This process involves the following activity: (1) glucose metabolism generating ATP; (2) subsequent closure of ATP-sensitive potassium (K_ATP_) channels leading to membrane depolarisation; (3) activation of voltage-gated calcium (CaV) channels resulting in a sharp rise in intracellular calcium ion (Ca^2+^) levels; and (4) Ca^2+^-dependent exocytosis of the readily releasable pool of insulin secretory granules.

Insulin secretion is modulated by hormones such as glucagon [5], glucagon-like peptide-1 (GLP-1) [6] and glucose-dependent insulinotropic polypeptide (GIP) [7]. These hormones potentiate insulin secretion via their stimulatory G-protein (Gs)-coupled receptors (GPCRs), which primarily activate adenylyl cyclase to increase cAMP levels [8]. The cAMP elevation subsequently activates protein kinase A (PKA). The K_ATP_ channel subunits Kir6.2 and SUR1, as well as exocytic proteins, are phosphorylated by PKA to enhance insulin secretion [9, 10].

PKA is a tetrameric holoenzyme consisting of two regulatory (R) subunits and two catalytic (C) subunits [11]. The functional properties of PKA holoenzymes are primarily determined by their R subunits, which include two classes (RI and RII) each having α and β isoforms. Based on R subunit composition, PKA is classified into four isoforms: type IA (RIα); type IIA (RIIα); type IB (RIβ); and type IIB (RIIβ). We previously reported that islet beta cells express two types of PKA, namely type IA and type IIB PKA [12]. Type IIB PKA is uniquely expressed in beta cells compared with other islet cell types. Thus, we hypothesise that type IIB PKA may function as the primary mediator of GPCR-potentiated insulin secretion. In this study, we employed RIIβ-knockout (KO) mice and isolated islets to assess insulin secretion both in vitro and in vivo. We also investigated the molecular mechanisms underlying beta cell dysfunction in RIIβ-KO islets by whole-cell patch-clamp recordings, RNA-seq and electron microscopy analysis.

## Methods

For detailed methods, please refer to electronic supplementary material (ESM) Methods.

### Animals

RIIβ-KO mice were generated using a DNA transposon *piggyBac* (PB) as described previously [13]. All animal handling protocols were approved by the Fudan University Animal Ethics Committees and followed the Chinese National Institute of Health guidelines on the care and use of animals.

### GTT and hormone measurements

For GTTs, fasted mice received d-glucose at 2.0 g/kg body weight (BW) by either i.p. injection (for IPGTT) or oral gavage (for OGTT). The blood collected from the orbital sinus of fasted mice under isoflurane anaesthesia was used for insulin, glucagon and somatostatin measurements.

### Mouse islet isolation and INS-1(832/13) cell culture

Pancreatic islets were isolated from mice using collagenase digestion and density gradient centrifugation as previously described [12]. INS-1(832/13) cells were maintained in RPMI 1640 containing 11.1 mmol/l glucose, 10% FBS and 50 µmol/l 2-mercaptoethanol. Transfection of siRNA was performed with Lipofectamine RNAiMAX.

### Insulin secretion assay in mice and in isolated islets

For in vivo studies, blood was drawn from the tail vein of mice at 0 and 10 min after glucose administration. Plasma was separated and used for insulin measurement. For in vitro studies, insulin release was measured using both static batch incubations and dynamic perifusion systems.

### Immunoblotting, quantitative PCR and RNA-sequencing analysis

Western blotting analysis was performed as described previously [14]. Total RNA was measured by quantitative real-time PCR (qPCR) or subjected to sequencing using Illumina BGIseq500 platform. Differentially expressed genes were detected with criteria set as *Q* value ≤0.05 or FDR ≤0.001. The antibodies and oligonucleotide primers are listed in ESM Tables 1, 2.

### Electron microscopy

Islets were pre-fixed in 2.5% glutaraldehyde overnight at 4℃ and then embedded in SPI-PON 812 resin. Images were captured using a transmission electron microscope (Tecnai G2 Spirit TWIN, FEI).

### Electrophysiology

Whole-cell patch-clamp technique was employed to record K_ATP_ and Ca^2+^ currents in single islet beta cells. K_ATP_ currents were recorded using perforated-patch recordings under both voltage step and voltage ramp protocols. Tolbutamide was employed to confirm K_ATP_ currents. Ca^2+^ currents were analysed as previously described [15]. The voltage dependence of the Na^+^ current inactivation was used to identify beta cells [16]. All electrophysiological recordings were performed at 30℃ using a feedback in-line heater. Data were filtered at 5 kHz and sampled at 10 kHz using an IPA Integrated Patch Amplifier with Igor Pro acquisition software.

### Statistical analysis

The results are expressed as mean ± SEM. For two-group datasets, analysis was done by unpaired two-tailed Student’s *t* test. For three or more independent groups of datasets, one-way ANOVA followed with Tukey corrected multiple comparison analysis was used. A *p* value <0.05 was considered statistically significant. Statistical analysis was performed using GraphPad Prism 8.2.1.

## Results

### RIIβ KO reduces the effects of GLP-1 on glucose tolerance

**Fig. 1:**
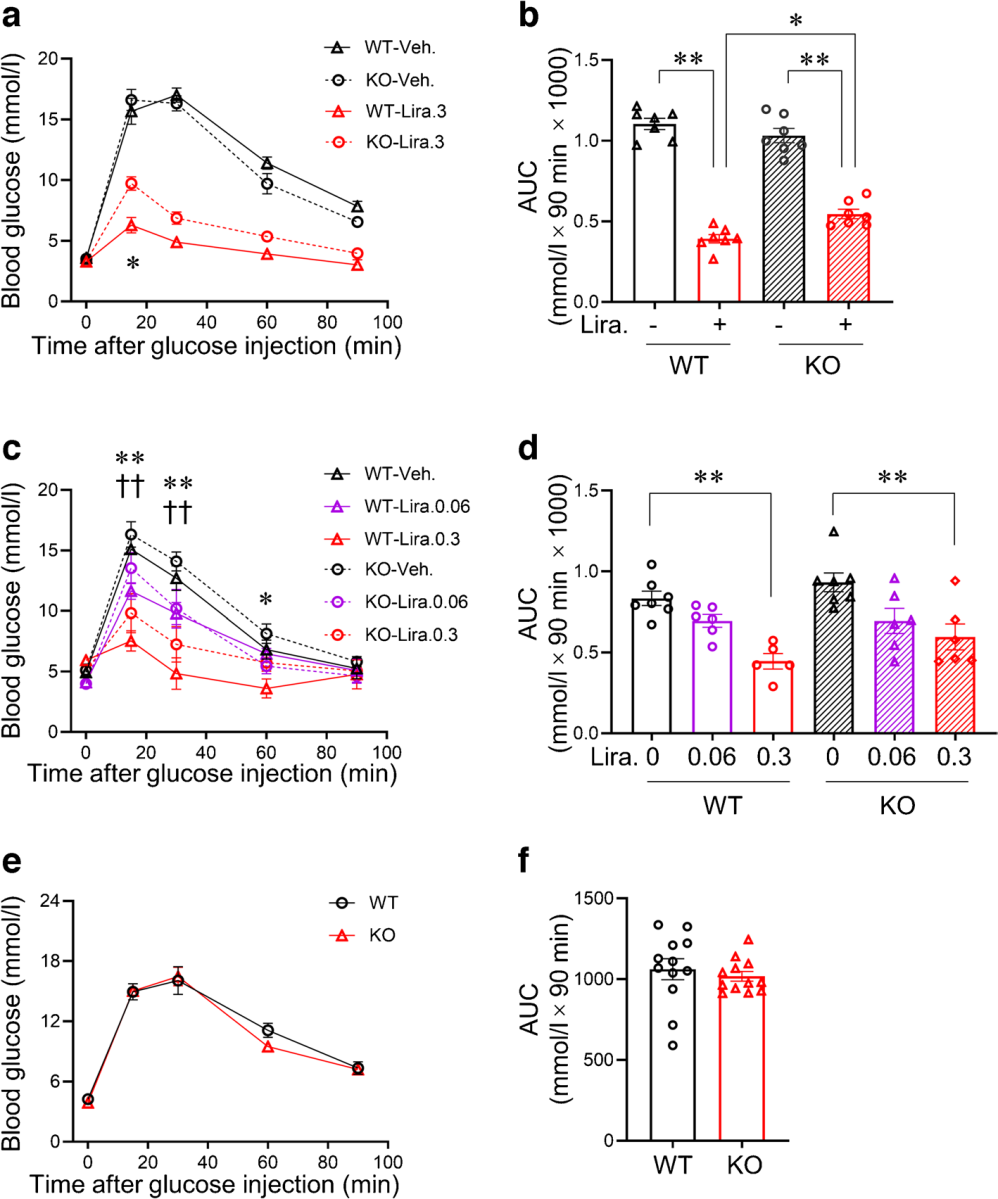
RIIβ KO reduces the effects of GLP-1 on glucose tolerance. (**a**) Blood glucose levels during an IPGTT. Liraglutide at a dose of 3 mg/kg BW was administered 30 min before glucose injection. *p<0.05, WT-Lira.3 vs KO-Lira.3; *n*=7 per group. (**b**) Glucose AUC of the IPGTT from data shown in (**a**). *p<0.05, **p<0.01; *n*=7 per group. (**c**) Blood glucose levels during an IPGTT. Liraglutide at dose of either 0.3 or 0.06 mg/kg BW was administered 30 min before glucose injection. *p<0.05, **p<0.01, WT-Lira.0.3 (*n*=5) vs WT-Vehicle (*n*=7); ^††^*p*<0.01 KO-Lira.0.3 (*n*=6) vs KO-Vehicle (*n*=7); *n*=6 other two groups. (**d**) Glucose AUC of the IPGTT from data shown in (**c**). **p<0.01; *n*=5 WT-Lira.0.3, *n*=7 WT-Vehicle, *n*=7 KO-Vehicle, *n*=6 other groups. (**e**) Blood glucose levels during an OGTT. *n*=12 per group. (**f**) Glucose AUC of the OGTT from data shown in (**e**). Data are mean ± SEM; one-way ANOVA followed by Tukey test (**a**–**d**) or unpaired *t* test (**e**, **f**). Lira., liraglutide; Veh., vehicle (saline [154 mmol/l NaCl])

GLP-1 regulates glucose metabolism mainly via activating Gs-coupled GLP-1 receptors, which further induces cAMP pathway [6]. Given that PKA is the primary downstream effector of cAMP, we investigated whether type IIB PKA plays a role in GLP-1’s effects. We employed a whole-body RIIβ-KO mouse and injected liraglutide at a dose of 3 mg/kg BW 30 min before initiation of an IPGTT. Liraglutide treatment significantly lowered blood glucose levels in wild-type (WT) mice, while this effect decreased in RIIβ-KO mice (Fig. 1a). Consistently, the AUC analysis of IPGTT showed that liraglutide treatment decreased the glucose excursion in both WT and RIIβ-KO mice with a reduced effect in RIIβ-KO mice (Fig. 1b). Treatment of mice with lower doses of liraglutide (0.3 and 0.06 mg/kg BW) showed a dose-dependent glucose-lowering effect (Fig. 1c). Under these conditions, liraglutide’s effects were moderately inhibited in RIIβ-KO mice. Consistent results were obtained from the IPGTT AUC analysis (Fig. 1d). Owing to the contribution made by incretin hormones to postprandial glucose metabolism [17, 18], we investigated whether type IIB PKA plays a role during an OGTT. No difference was observed in glucose levels between WT and RIIβ-KO mice (Fig. 1e, f). These results suggest that type IIB PKA plays a critical role in GLP-1’s effects on systemic glucose metabolism.

### RIIβ KO inhibits the effects of GLP-1 on glucose-induced insulin secretion

**Fig. 2:**
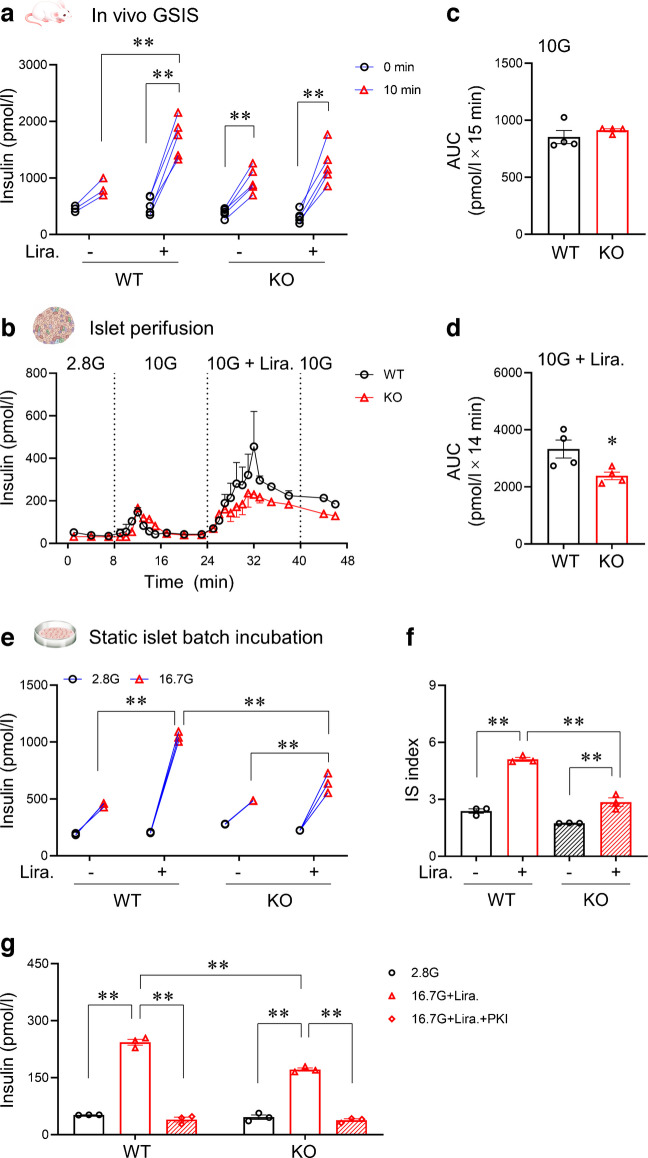
RIIβ KO inhibits the promotion of GLP-1 on GSIS. (**a**) Serum insulin levels before and 10 min after glucose injection in mice. *n*=3 WT-Vehicle, *n*=5 other groups. (**b**) Insulin secretion profile from isolated islets during in vitro islet perifusion assay. Islets were perifused sequentially with 2.8 mmol/l glucose (2.8G), 10 mmol/l glucose (10G), 10 mmol/l glucose with liraglutide (50 nmol/l, 10G + Lira.), and 10 mmol/l glucose (10G). (**c**, **d**) Insulin AUC of islets treated with 10G (**c**) or 10G + Lira. (**d**) from data shown in (**b**). *n*=4 per group (**b**, **c**, **d**). (**e**) GSIS from isolated islets during in vitro static batch incubations at 2.8 mmol/l glucose (2.8G) and 16.7 mmol/l glucose (16.7G). (**f**) Insulin secretion index from data shown in (**e**). *n*=3 per group (**e**, **f**). (**g**) Insulin secretion from isolated islets treated without or with liraglutide (50 nmol/l) in the presence or absence of PKA inhibitor during in vitro static insulin secretion assay. *n*=3 per group. Data are mean ± SEM; *p<0.05, **p<0.01; one-way ANOVA followed by Tukey test (**a**, **e**, **f**, **g**) or unpaired *t* test (**c**, **d**). IS, insulin secretion; Lira., liraglutide; PKI, PKA inhibitor

The primary metabolic action of GLP-1 is to promote insulin secretion [18]. We therefore investigated the involvement of type IIB PKA in this process. We observed that glucose injection increased serum insulin levels and liraglutide treatment further promoted this increase in WT mice (Fig. 2a). RIIβ-KO mice exhibited a comparable increase in GSIS to WT mice, but a moderate (not statistically significant) additional increase in response to liraglutide (Fig. 2a). In addition, we measured other islet-specific hormones under fasting conditions. With comparable fasting blood glucose levels, RIIβ-KO mice had lower serum levels of glucagon and somatostatin than WT mice (ESM Fig. 1a–g).

We then isolated islets from mice and measured their dynamic insulin secretion with a perifusion system. Insulin content in RIIβ-KO islets was comparable with that in WT islets (ESM Fig. 1h). Glucose treatment gradually increased insulin levels, with no significant difference between WT and RIIβ-KO islets (Fig. 2b). The liraglutide-treated WT islets exhibited an additional increase in insulin secretion, while RIIβ-KO islets showed a reduced response to liraglutide. The AUC analysis of islet perifusion confirmed a significantly attenuated liraglutide-induced secretory response in RIIβ-KO islets (Fig. 2c, d). Furthermore, we measured the insulin secretion under a static culture condition. Consistently, WT islets and RIIβ-KO islets showed comparable GSIS, while RIIβ-KO islets had a reduced secretory response to liraglutide (Fig. 2e, f). When we blocked the PKA activation by GLP-1 with a PKA inhibitor, both WT and RIIβ-KO islets showed similar insulin level (Fig. 2g). These results indicate that type IIB PKA mediates GLP-1’s effects on insulin secretion.

### RIIβ KO blocks the PKA signalling activation by GLP-1 in islets

**Fig. 3:**
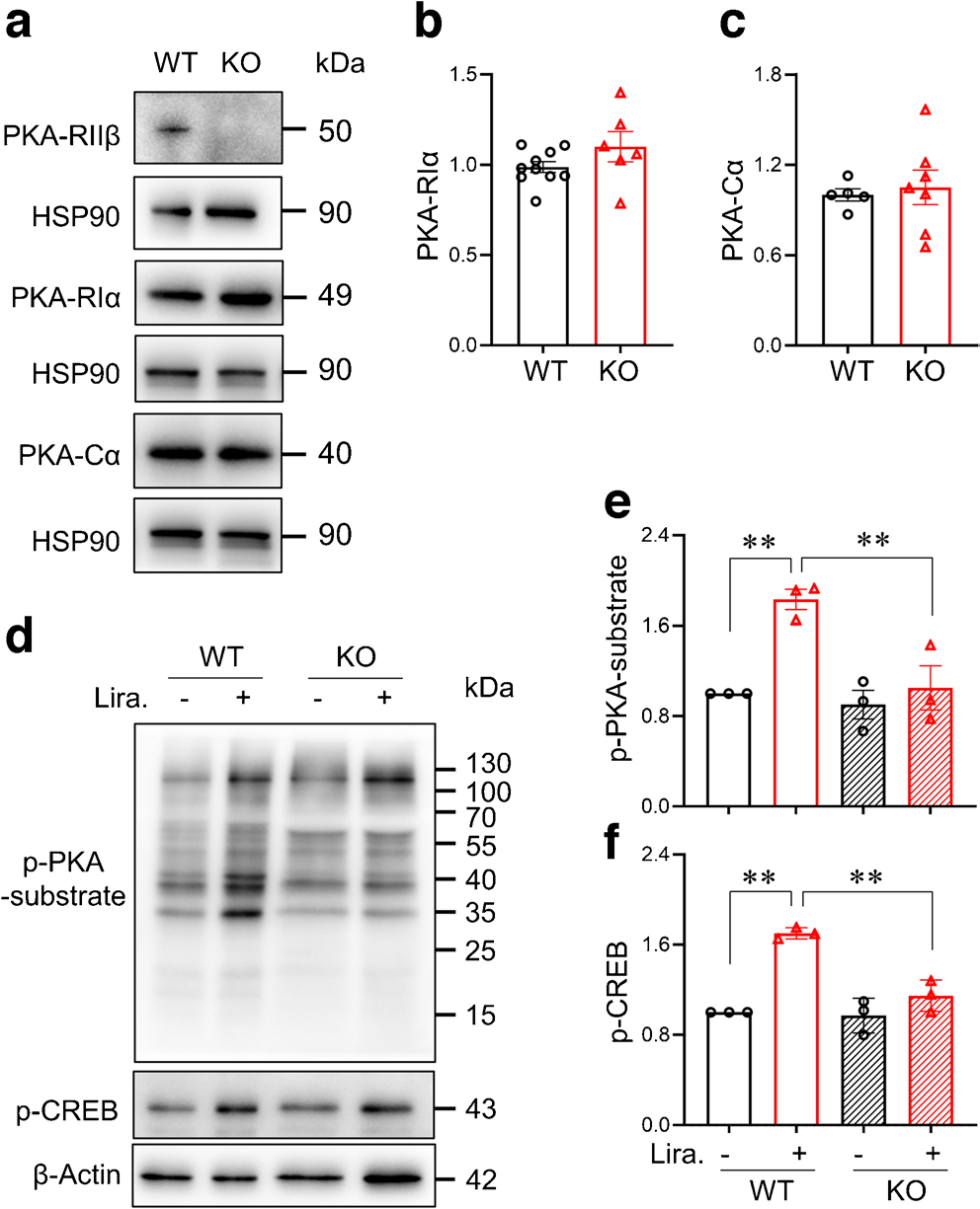
RIIβ KO blocks the activation of PKA signalling by GLP-1 in islets. (**a**) Western blotting analysis of the PKA subunit expression in islets isolated from WT and RIIβ-KO mice. (**b**) Quantitative analysis of PKA-RIα subunit expression. *n*=10 WT, *n*=6 RIIβ KO. (**c**) Quantitative analysis of PKA-Cα subunit expression. *n*=5 WT, *n*=7 RIIβ KO. (**d**) Western blotting analysis of the phospho-PKA substrate levels in isolated islets treated without or with liraglutide. (**e**) Quantitative analysis of phospho-PKA substrate level. *n*=3 per group. (**f**) Quantitative analysis of phospho-CREB. *n*=3 per group. Data are mean ± SEM. **p<0.01, unpaired *t* test (**b**, **c**) or one-way ANOVA followed by Tukey test (**e**, **f**). Lira., liraglutide

With the notion that islet beta cells have both type IA and type IIB PKA [12], we investigated the expression level of PKA subunits in RIIβ-KO islets (Fig. 3a). We observed that WT islets and RIIβ-KO islets had comparable levels of RIα and Cα, besides the absence of RIIβ in RIIβ-KO islets (Fig. 3b, c). Then, we attempted to test the activation of PKA signalling in response to liraglutide by measuring the level of phospho-PKA substrate. The isolated islets were cultured in media without FBS for 2 h to establish a quiescent state. Liraglutide treatment for 20 min significantly increased the level of phospho-PKA substrate and phospho-CREB in WT islets, while this effect was significantly attenuated in RIIβ-KO islets (Fig. 3d–f). These results demonstrate that the impaired insulin secretory response in RIIβ-KO islets primarily stems from the specific ablation of type IIB PKA, without the compensatory involvement of type IA PKA.

### RIIβ KO reduces the effects of insulin secretagogue hormones

**Fig. 4:**
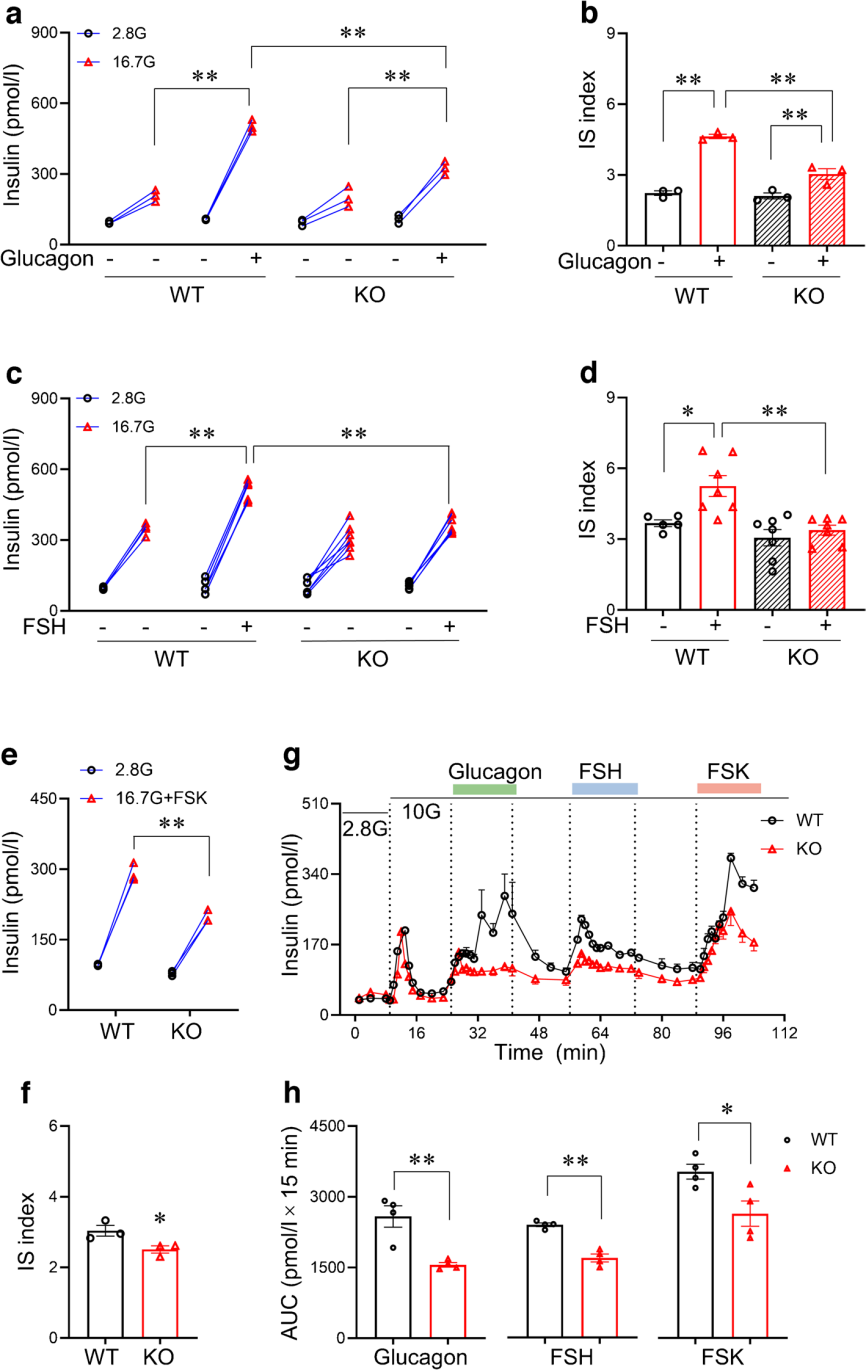
RIIβ KO reduces the effects of insulin secretagogue hormones. (**a**) GSIS from isolated islets treated without or with glucagon (10 nmol/l) during in vitro static batch incubations with 2.8 mmol/l glucose (2.8G) and 16.7 mmol/l glucose (16.7G). (**b**) Insulin secretion index from data shown in (**a**). *n*=3 per group (**a**, **b**). (**c**) GSIS from isolated islets treated without or with FSH (10 U/l), during in vitro static batch incubations. *n*=5 WT-2.8G, *n*=5 WT-16.7G, *n*=7 other groups. (**d**) Insulin secretion index from data shown in (**c**). *n*=5 WT-16.7G, *n*=7 WT-16.7G-FSH, *n*=5 KO-16.7G, *n*=7 KO-16.7G-FSH (**c**, **d**). (**e**) Insulin secretion from isolated islets treated without or with FSK (5 μmol/l) during in vitro static batch incubations. (**f**) Insulin secretion index from data shown in (**e**). *n*=3 per group (**e**, **f**). (**g**) Insulin secretion profile from isolated islets during in vitro islet perifusion assay. Islets were perifused sequentially with 2.8G, 10 mmol/l glucose (10G), 10G with glucagon (10 nmol/l), 10G with FSH (10 U/l), and 10G with FSK (5 μmol/l). (**h**) Insulin AUC of the islets treated with 10G-Glucagon, 10G-FSH and 10G-FSK from data shown in (**g**). Data are mean ± SEM. *p<0.05, **p<0.01, one-way ANOVA followed by Tukey test (**a**–**e**) or unpaired *t* test (**f**, **h**). IS, insulin secretion

Besides GLP-1, insulin secretion is regulated by several hormones via targeting Gs–cAMP–PKA signalling. One example is glucagon secreted from islet alpha cells which exerts a paracrine effect on beta cells by increasing insulin secretion [19]. Glucagon treatment promoted GSIS in WT islets, while this effect was attenuated in RIIβ-KO islets (Fig. 4a, b). Another example is follicle-stimulating hormone (FSH), which is reported to regulate insulin secretion in a PKA-dependent manner [20]. FSH treatment increased GSIS in WT islets, but had no effect in RIIβ-KO islets (Fig. 4c, d). To further confirm the specific contribution of type IIB PKA signalling, islets were treated with forskolin (FSK), a direct adenylate cyclase activator. We observed that FSK treatment triggered a significant increase in insulin secretion from WT islets but this effect was attenuated in KO islets (Fig. 4e, f). We then traced the dynamic of insulin secretion in islets treated sequentially with glucagon, FSH and FSK. In line with the static incubation results, the amplitude of insulin secretion with all treatments decreased in RIIβ-KO islets (Fig. 4g). AUC analysis further confirmed that RIIβ-KO islets secreted less insulin in response to all stimuli compared with WT islets (Fig. 4h). These results suggest that type IIB PKA is a primary and common pathway for insulin secretagogue hormones that target Gs-coupled receptors.

### RIIβ KO reduces basal K_ATP_ channel activity and blocks liraglutide-mediated channel inhibition

**Fig. 5:**
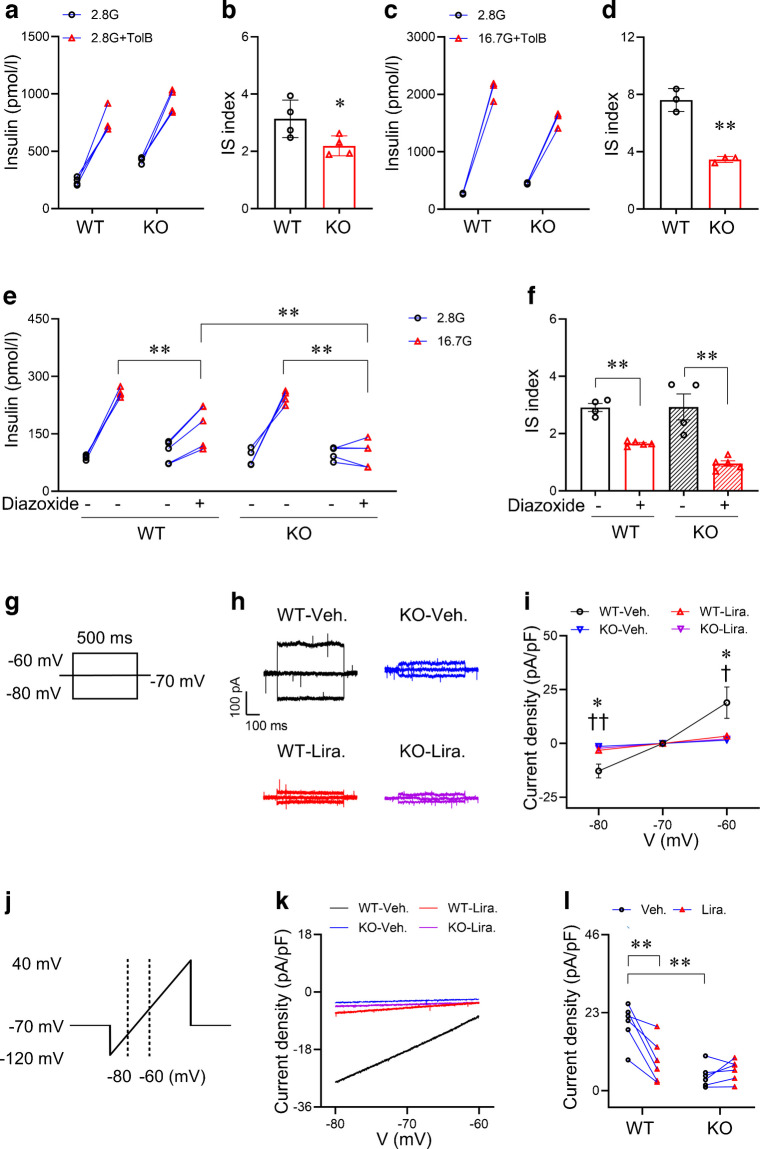
RIIβ-KO reduces basal K_ATP_ channel activity and blocks liraglutide-mediated channel inhibition. (**a**) Insulin secretion from isolated islets at 2.8 mmol/l glucose (2.8G) treated without or with tolbutamide (0.5 mmol/l) during in vitro static insulin secretion assay. (**b**) Insulin secretion index from data shown in (**a**). *p<0.05; *n*=4 per group (**a**, **b**). (**c**) Insulin secretion from isolated islets at 16.7 mmol/l glucose (16.7G) treated with tolbutamide during in vitro static batch incubations. (**d**) Insulin secretion index from data shown in (**c**). **p<0.01; *n*=3 per group (**c**, **d**). (**e**) GSIS from isolated islets treated without or with diazoxide (0.25 mmol/l) during in vitro static batch incubations. (**f**) Insulin secretion index from data shown in (**e**). **p<0.01; *n*=4 WT-16.7G, *n*=5 WT-16.7G-diazoxide, *n*=4 KO-16.7G, *n*=5 KO-16.7G-diazoxide (**e**, **f**). (**g**) Schematic diagram of perforated-patch recordings with voltage step protocol from −80 mV to −60 mV. (**h**) Representative traces of K_ATP_ currents recorded in WT and RIIβ-KO beta cells before and after liraglutide (100 nmol/l) treatment. (**i**) The K_ATP_ current density relative to the level at −70 mV. *p<0.05, WT-Lira. vs WT-Vehicle; ^†^*p*<0.05, ^††^*p*<0.01, KO-Vehicle vs WT-Vehicle; *n*=6 per group. (**j**) Schematic diagram of perforated-patch recordings with voltage ramp protocol. (**k**) Representative traces of whole-cell K_ATP_ currents ranging from −80 mV to −60 mV recorded in WT and RIIβ-KO beta cells before and after liraglutide (100 nmol/l) treatment. (**l**) Changes in K_ATP_ current density between −80 mV and −60 mV. **p<0.01; *n*=6 per group. The validation of K_ATP_ current is shown in ESM Fig. 2. Data are mean ± SEM; one-way ANOVA followed by Tukey test (**a**, **c**, **e**, **f**, **i**, **l**) or unpaired *t* test (**b**, **d**). IS, insulin secretion; Lira., liraglutide; TolB, tolbutamide; Veh., vehicle (extracellular solution for K_ATP_ current recording)

The K_ATP_ channel is a key mediator of GSIS and a known phosphorylation target of PKA [9, 10]. Sulfonylureas are a class of glucose-lowering medications that stimulate insulin secretion by targeting K_ATP_ channels in pancreatic beta cells [21]. To investigate the role of K_ATP_ channels, we modulated their activity using pharmacological agents during a GSIS assay. We observed that treatment with the K_ATP_ inhibitor tolbutamide increased insulin secretion at low glucose level in WT islets but this effect was blunted in RIIβ-KO islets (Fig. 5a, b). Similarly, tolbutamide’s promotor effects under high glucose condition were reduced in RIIβ-ablated islets (Fig. 5c, d). Conversely, the K_ATP_ activator diazoxide suppressed GSIS in both WT and RIIβ-KO islets (Fig. 5e, f).

We next directly measured K_ATP_ channel activity in islet beta cells using perforated-patch recordings, as this configuration is the preferred approach for probing the effects mediated by soluble small molecules such as cAMP [22, 23]. Beta cells were identified by the voltage dependence of the Na^+^ current inactivation. Using a voltage step protocol, K_ATP_ currents were quantified within the range of −80 mV to −60 mV (Fig. 5g–i). This voltage range was selected because K_ATP_ currents are prominent and minimally contaminated by other voltage-gated currents [24]. In WT beta cells, K_ATP_ currents exhibited a voltage-dependent change, whereas RIIβ ablation decreased this change. Liraglutide treatment significantly inhibited the K_ATP_ current change, while this inhibitory effect was blocked by RIIβ ablation. We also measured K_ATP_ currents using a voltage ramp protocol, and observed consistent effects of RIIβ KO and liraglutide on K_ATP_ currents (Fig. 5j–l). To validate that the recorded currents were mediated by K_ATP_, we performed pharmacological characterisation under this configuration. The currents were suppressed by 0.1 mmol/l tolbutamide (ESM Fig. 2). These results demonstrate that RIIβ KO directly inhibits K_ATP_ channel activity, which contrasts with its role in mediating GLP-1’s stimulatory effects on insulin secretion, suggesting a complex effect of PKA on insulin secretion.

### RIIβ-ablated islets show reduced L-type Ca^2+^ currents in response to GLP-1 stimulation

**Fig. 6:**
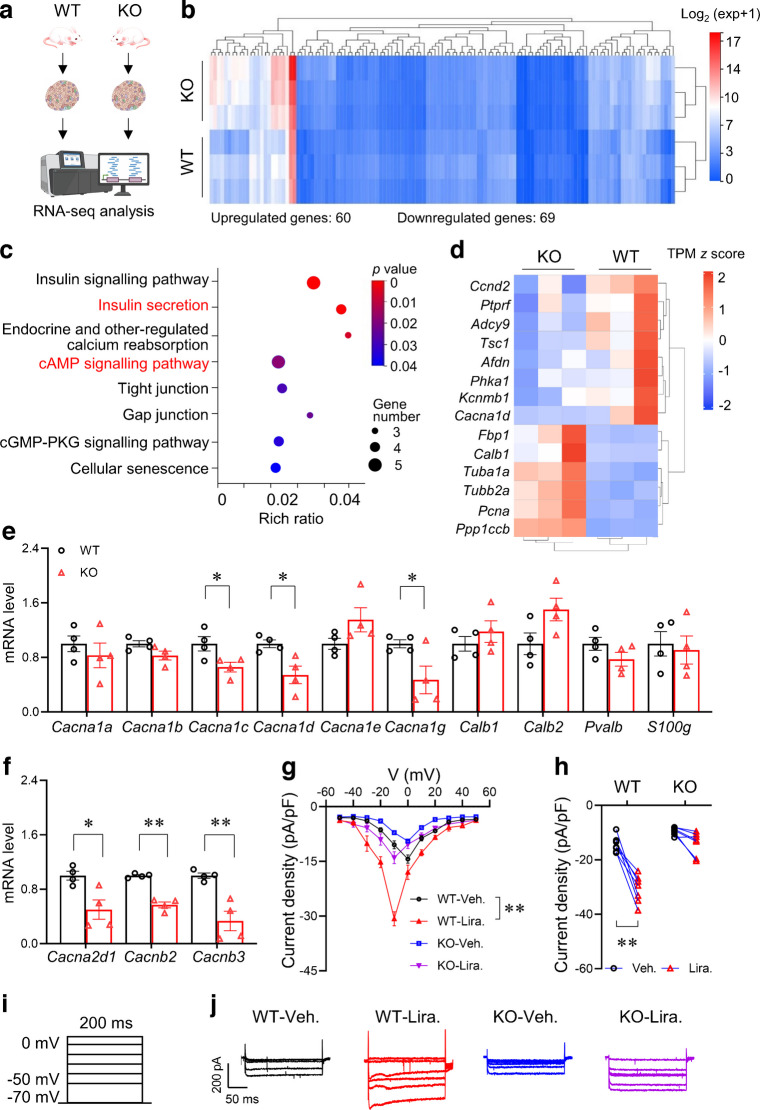
RIIβ-ablated islets show reduced L-type Ca^2+^ currents in response to GLP-1 stimulation. (**a**) Schematic diagram of RNA-seq analysis. Islets were isolated from WT and RIIβ-KO mice, hand-picked and processed for RNA extraction and subsequent sequencing analysis. (**b**) Heatmap of 129 differential genes of RIIβ-KO islets compared with WT islets. (**c**) KEGG enrichment analysis based on differential genes. *n*=3 per group (**b**, **c**). (**d**) Insulin secretion gene expression from RNA-seq data. (**e**) Quantitative PCR analysis of CBP genes in isolated islets. *n*=4 per group. (**f**) Quantitative PCR analysis of isolated islets of CaV genes. *n*=4 per group. (**g**–**j**) Whole-cell Ca^2+^ currents were recorded in WT and RIIβ-KO beta cells before and after liraglutide (100 nmol/l) treatment. (**g**) The I/V curve of the Ca^2+^ current density between −50 mV and 50 mV. (**h**) Changes in maximum Ca^2+^ current density before and after liraglutide treatment. *n*=7 per group (**g**, **h**). (**i**) Schematic diagram of voltage clamp recordings of Ca^2+^ current. (**j**) Representative traces of Ca^2+^ current recordings. Data are mean ± SEM; *p<0.05, **p<0.01, unpaired *t* test (**e**, **f**) or one-way ANOVA followed by Tukey test (**g**, **h**). Lira., liraglutide; TPM, transcripts per million; Veh., vehicle (extracellular solution for Ca^2+^ current recording)

To elucidate the complex effects of RIIβ ablation on the insulin secretion pathway, we performed RNA-seq of isolated islets (Fig. 6a). Comparative analysis revealed 129 differentially expressed genes (Fig. 6b). Kyoto Encyclopedia of Genes and Genomes (KEGG) pathway analysis identified the top eight enriched pathways, including the insulin secretion and cAMP signalling pathways (Fig. 6c). We next analysed the insulin secretion genes and observed decreased expression of CaV channel gene (*Cacna1d*), along with increased expression of Ca^2+^-binding protein (CBP) gene (*Calb1*) and cell proliferation marker gene (*Pcna*) in RIIβ-ablated islets (Fig. 6d).

As a primary driver of insulin granule exocytosis [25, 26], Ca^2+^ homeostasis is tightly controlled by CaV channels and CBPs. CaV channels are multi-subunit complexes composed of five subunits: the pore-forming α1 subunit; and accessory α2, β, δ and γ subunits. Pancreatic beta cells express multiple CaV channel genes, including those encoding the α_1_ subunit (*Cacna1c*, *Cacna1d*, *Cacna1a*, *Cacna1b*, *Cacna1e*, *Cacna1g*), β subunit (*Cacnb2*, *Cacnb3*) and α2δ subunit (*Cacna2d1*) [27]. CBPs control Ca^2+^ signalling by free Ca^2+^ sequestration [28]. In pancreatic beta cells, the major cytosolic CBPs include calretinin (encoded by *Calb2*), calbindin-D9K (*S100g*), calbindin-D28K (*Calb1*) and parvalbumin-β (*Pvalb*) [29]. To further analyse the expression profiles of CaV channels and CBPs, we performed qPCR on isolated islets. In line with RNA-seq results, *Cacna1d* expression was decreased in RIIβ-KO islets (Fig. 6e). Meanwhile, other CaV subunit genes (*Cacna1c*, *Cacna1g*, *Cacna2d1*, *Cacnb2* and *Cacnb3*) also decreased in RIIβ-KO islets (Fig. 6e, f). However, the downregulation of the CBP gene expression detected by RNA-seq was not confirmed by qPCR (Fig. 6e). These results suggest that reduced CaV channel expression may account for impaired insulin secretion in RIIβ-KO islets.

To test the functional consequence of decreased CaV gene expression, we measured Ca^2+^ channel activity using whole-cell patch-clamp recordings. Ca^2+^ currents were isolated by adding the Na^+^ channel inhibitor tetrodotoxin and the K^+^ channel inhibitor 4-aminopyridine. Both WT and RIIβ-KO beta cells exhibited a typical voltage-dependent Ca^2+^ current profile (Fig. 6g). There was a subtle decrease of the Ca^2+^ current in RIIβ-KO beta cells compared with WT beta cells. Liraglutide treatment significantly stimulated Ca^2+^ current across most voltages in WT beta cells, consistent with previous reports [30]. Besides, liraglutide caused a leftward shift of the peak current voltage. When quantifying maximal current density, liraglutide treatment induced a higher maximum Ca^2+^ current density in WT beta cells but not RIIβ-KO beta cells (Fig. 6h–j). These results demonstrated that RIIβ ablation blocked the stimulation of Ca^2+^ channel by liraglutide. Taken together, these data suggest that Ca^2+^ channels are the key targets of type IIB PKA in mediating GLP-1’s effects.

### Altered insulin granule morphology contributes to the impaired response to GLP-1 in RIIβ-ablated beta cells

**Fig. 7:**
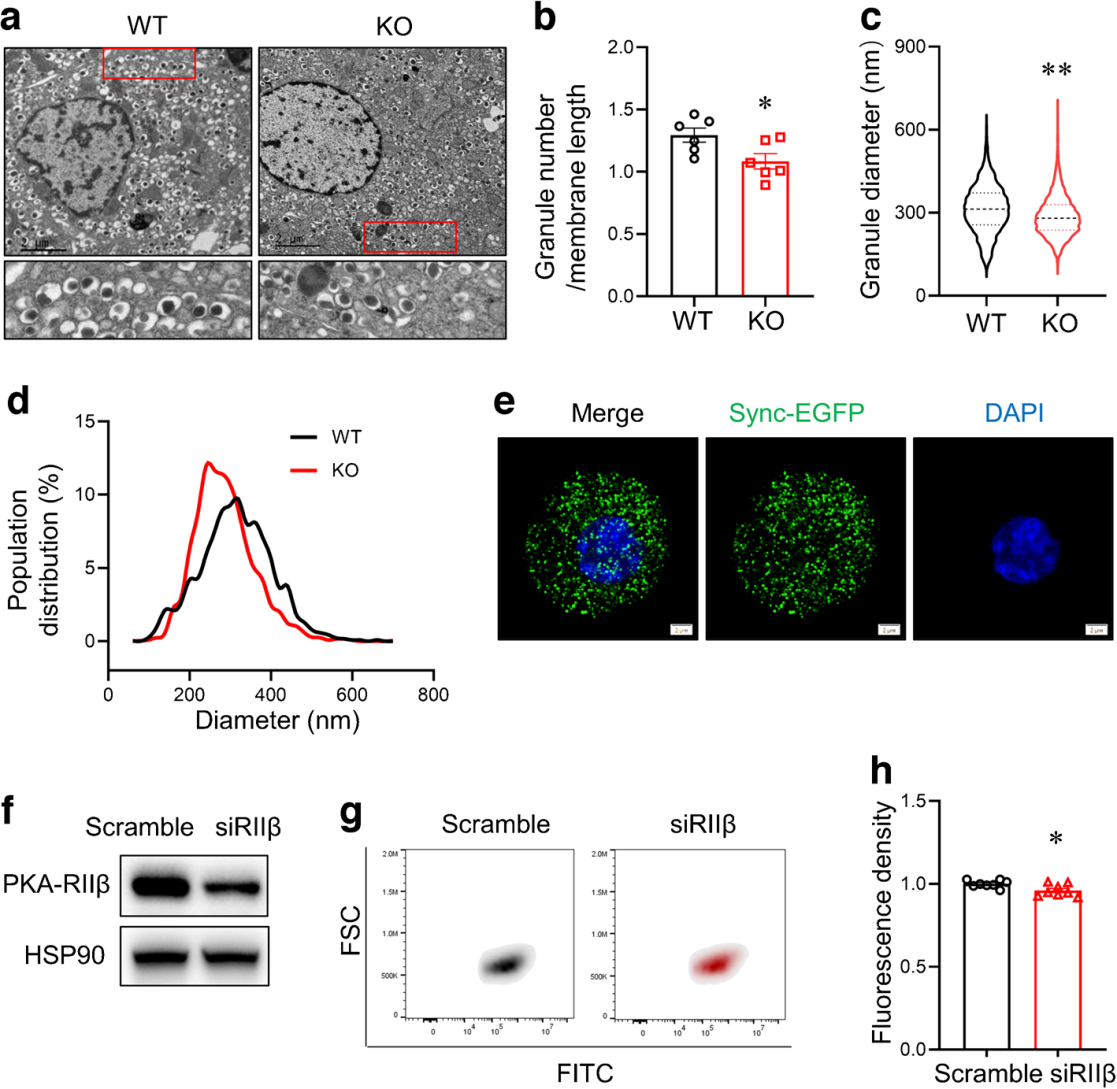
Altered insulin granule morphology contributes to the impaired insulin secretion in RIIβ-KO beta cells. (**a**–**d**) Representative electron microscopy images of islet beta cells (**a**) (scale bar, 2 μm) and quantification of the insulin granule near plasma membrane (**b**), diameter (**c**) and insulin granule diameter profile (**d**) from these images. *n*=6 per group (**b**, **d**); *n*=2644 WT, *n*=2250 RIIβ KO (**c**). (**e**) Representative images of dispersed mouse islet cells isolated infected with adenovirus carrying Syncollin-EGFP (Sync-EGFP). Scale bar, 2 μm. (**f**) Western blotting analysis of PKA RIIβ expression in INS-1(832/13) cells transfected with siRNA against RIIβ. (**g**, **h**) Fluorescence density (**g**) and quantification (**h**) in INS-1(832/13) cells transfected with adenovirus carrying Syncollin-EGFP. *n*=8 per group (**h**). Data are mean ± SEM; *p<0.05, **p<0.01, unpaired *t* test (**b**, **c**, **h**)

Given that the docked insulin granules represent the readily releasable pool and are the key target of PKA in the process of insulin secretion [31], we characterised the insulin granule morphology using an electron microscope. A significant decrease in the number of docked granules, defined as those within 100 nm of the plasma membrane, was observed in RIIβ-KO beta cells (Fig. 7a, b). Meanwhile, the granule size was reduced in RIIβ-KO beta cells, with a shift towards smaller granule populations (Fig. 7c, d). To further assess granule abundance, we infected isolated islets with adenovirus carrying Syncollin-EGFP (Fig. 7e). Due to the high variability in granule number across primary islet cells, we instead quantified the granules using flow cytometry with INS-1(832/13) cells. The cells infected with Syncollin-EGFP overexpression virus were further transfected with RIIβ-targeting siRNA, which markedly reduced RIIβ protein levels (Fig. 7f). Flow cytometry showed a broad distribution of EGFP-positive cells, alongside a 4% (significant) decrease in fluorescent density in RIIβ-knockdown cells (Fig. 7g, h). Taken together with the reduced Ca^2+^ current, these ultrastructural alterations demonstrate that RIIβ deletion disrupted both the granule priming and fusion steps of insulin secretion, suggesting type IIB PKA as a critical regulator of beta cell exocytosis.

## Discussion

In the present study, we demonstrated that type IIB PKA acted as a primary mediator of Gs-coupled receptor-potentiated insulin secretion. Genetic ablation of the RIIβ subunit in islet beta cells reduced the effects of GLP-1, glucagon and FSH on insulin secretion, as demonstrated by both dynamic islet perifusion studies and static islet batch incubations. With these beta cell autonomous defects, RIIβ-KO mice exhibited impaired glucose-lowering responses to GLP-1 during an IPGTT. We further identified the molecular mechanisms underlying beta cell dysfunction in RIIβ-KO islets, including altered K_ATP_ channel activity, diminished Ca^2+^ currents and abnormal insulin granule morphology. These findings revealed that type IIB PKA serves as an essential signalling hub for GPCR-mediated potentiation of insulin secretion.

Pancreatic beta cells express a diverse repertoire of GPCRs that are critical in sustaining adequate insulin secretion to ensure euglycaemia [8]. GPCRs share similar activation mechanisms. Ligand binding specifically triggers receptor conformational changes and subsequent heterotrimeric G-protein binding. The primary G-protein pathway classically associated with GLP-1 and glucagon is the Gs–cAMP–PKA pathway. Interestingly, FSH receptors exhibit the potential to activate G-protein signalling in a pleiotropic manner [32], mediating FSH-regulated GSIS in a bell curve manner [20]. PKA acts as the main effector of cAMP signals from hundreds of GPCRs, regulating cell metabolism, growth and survival [33]. Overexpression of the PKA C subunit increases PKA activity and enhances insulin secretion [34, 35]. Interestingly, PKA does not fully mediate GLP-1-induced secretory response. An alternative cAMP-dependent but PKA-independent pathway, controlled by the exchange protein directly activated by cAMP 2 (EPAC2) [36], has been identified. Consistent with this pathway divergence, our study showed that RIIβ ablation selectively blocked the PKA-dependent pathway, resulting in a partial, but not complete, attenuation of GLP-1’s effects on both GSIS and IPGTT.

We previously identified two types of PKA regulatory subunits in pancreatic beta cells. Genetic ablation of RIα vs RIIβ produces differential phenotypic consequences. PKA RIα ablation in beta cells does not alter fasting insulin levels but selectively augments GSIS, resulting in improved glucose tolerance [37]. This phenotype is recapitulated in humans harbouring inactivating mutations in the RIα-coding gene *Prkar1a* (Carney’s complex) [38, 39]. In contrast, PKA RIIβ ablation in beta cells, as demonstrated in this study, decreased basal fasting insulin levels and specifically inhibited GLP-1’s potentiating effects on insulin secretion. These differential effects may arise from distinct PKA activity after regulatory subunit deletion. It is likely that the ablation of the predominant regulatory subunit will eliminate its constitutive inhibition of the catalytic subunit, thereby enhancing PKA activity. Alternatively, deletion of the non-predominant regulatory subunit will induce compensatory recruitment of catalytic subunits by the predominant regulatory subunits, consequently eliminating the specific signalling mediated by the deleted PKA isoform. In line with this notion, RIIβ ablation in adipose tissues and brain tissue where it is the predominant regulatory subunit increases PKA activity and activates its downstream pathway [40–42]. This framework consequently explains the observations in pancreatic beta cells. Given that beta cells have more RIα than RIIβ [12], RIα ablation induces the disinhibition of catalytic subunits and subsequent enhancement of PKA activity, whereas RIIβ deletion eliminates effects specifically mediated by type IIB PKA.

The effects of PKA on first-phase insulin release lie downstream of Ca^2+^ signalling and plasma membrane depolarisation, as evidenced by overexpression of PKA catalytic subunit in pancreatic beta cells [34]. Further, studies using RIα deletion mice reveal that PKA signalling increases the number and size of docked insulin vesicles [37]. Consistent with these studies, we showed that type IIB PKA ablation decreased the size distribution of mature insulin vesicles and docked granule density. A number of proteins have been identified as PKA kinase targets, including SNAP25, tomosyn-1, tomosyn-2, snapin, syntaxin-4 and cysteine string protein. Type II PKA is anchored to mature insulin secretory granules [43], supporting the hypothesis that PKA potentiates acute-phase GSIS by direct phosphorylation of protein components of the insulin secretory machinery.

PKA regulates insulin secretion through multiple distinct mechanisms. Our study showed altered K_ATP_ channel activity without corresponding gene expression changes, suggesting its post-translational regulation by PKA phosphorylation. Multiple phosphorylation sites have been identified as the targets of PKA within the K_ATP_ subunits SUR1 and Kir6.2. The phosphorylation levels at Kir6.2(S372) and SUR1(S1448) are regulated by Gs signalling, whereas SUR1(S1571) phosphorylation is not. Furthermore, phosphorylation at these distinct sites differentially regulates K_ATP_ channel activity. Specifically, phosphorylation of Kir6.2(S372) increases channel activity. In contrast, phosphorylation of SUR1(S1448) exerts either inhibitory or stimulatory effects on the channel, dependent on the cellular ATP/ADP level. However, the functional significance of these sites remains to be validated, given their localisation distal to canonical functional domains. Bioinformatics analysis predicts multiple PKA phosphorylation sites on SUR1 and Kir6.2, distributed across various regions. Our study revealed a critical role of type IIB PKA in K_ATP_ channel regulation, suggesting that phosphorylation at one or more of these sites underlies the observed functional modulation.

Beyond direct phosphorylation, PKA also regulates insulin secretion by modulating gene transcription. We demonstrated here that the expression level of genes coding for CaV channels decreased in RIIβ-KO islets. The role of CaV channels in insulin secretion has been comprehensively reviewed by Yang and Berggren [27]. While recent studies have implicated additional contributions from other types of CaV channels [44, 45], consensus confirms CaV1.2 as the major type that plays a predominant role in Ca^2+^-triggered insulin secretion. Pancreatic beta cell-selective ablation of CaV1.2 by *Cacna1c* knockout abolishes insulin secretion and results in systemic glucose intolerance in mice [15]. We observed decreased expression of *Cacna1c* in RIIβ-KO islets, consistent with the subsequent findings of reduced Ca^2+^ currents in response to GLP-1 stimulation.

GLP-1 is postprandially secreted by intestinal L cells to enhance insulin secretion from pancreatic beta cells. Luminal nutrient sensing directly stimulates L cell secretion of GLP-1 [17]. Given that intestinal glucose consistently triggers GLP-1 secretion, OGTT is an adequate tool for examining postprandial GLP-1 response [46]. The incretin hormones GLP-1 and GIP together account for 50–70% of insulin secretion during an OGTT [18]. In this study, although exogenously administered GLP-1 during IPGTT showed attenuated effects in our model, glucose tolerance remained unaltered during an OGTT. Notably, distinct GLP-1 response patterns between IPGTT and OGTT have been documented [47]. This divergence aligns with two key physiological observations. First, GLP-1-receptor-KO mice display glucose intolerance with impaired insulin increase only to a large glucose load (75 and 100 mg) but maintain normal glucose tolerance at smaller glucose loads (25 and 50 mg) [48]. Second, and critically, human ex vivo studies establish that glucose concentrations ≥200 mmol/l are required to stimulate significant GLP-1 secretion from ileal cultures [49]. Thus, the null effect of RIIβ KO during OGTT likely reflects the subthreshold activation of endogenous GLP-1 pathways under these experimental conditions.

While the pleiotropic actions of PKA currently limit its direct therapeutic potential, distinguishing the functional specificity of individual PKA isoforms remains critical for metabolic regulation research. This study fundamentally advances our understanding of endocrine signal transduction by demonstrating how a common second messenger achieves pathway selectivity through discrete PKA holoenzyme populations. We also established the critical role of type IIB PKA in GLP-1’s insulinotropic effects, revealing new insights into cAMP–PKA signalling specificity in pancreatic beta cells. These findings may eventually guide the development of PKA-isoform-selective modulators to enhance GLP-1-based therapies for diabetes.

## Supplementary Information

Below is the link to the electronic supplementary material.

ESM (PDF 402 KB)

## Data Availability

The RNA-seq datasets have been submitted to NCBI Sequence Read Archive (SRA) and can be accessed via the following link: https://www.ncbi.nlm.nih.gov/sra/?term=SRX29481113.

## Abbreviations

BW: Body weight
CaV: Voltage-gated calcium (channel)
CBP: Calcium-binding protein
FSH: Follicle-stimulating hormone
FSK: Forskolin
GIP: Glucose-dependent insulinotropic polypeptide
GLP-1: Glucagon-like peptide-1
GPCR: G-protein-coupled receptor
Gs: Stimulatory G protein
GSIS: Glucose-stimulated insulin secretion
K_ATP_: ATP-sensitive potassium (channel)
KO: Knockout
PKA: Protein kinase A
qPCR: Quantitative real-time PCR
WT: Wild-type
