# Supplementary material for: Type IIB PKA serves as the primary effector of Gs-coupled receptor-potentiated insulin secretion in mice by orchestrating ion channels and granule phenotype
Source: Diabetologia. 2026 Jun 23;69(9):2585–98. doi: 10.1007/s00125-026-06781-8 (PMC13424381; doi:10.1007/s00125-026-06781-8)
Supplement: Supplementary file 1 — ESM (PDF 402 KB) [file 125_2026_6781_MOESM1_ESM.pdf]

Electronic Supplementary Material (ESM)

ESM Methods

2.1 Animals

RIIβ KO mice (Prkar2b$^{PB/PB}$; strain number 080527004-HRA/PB) were generated on the FVB/NJ background using a DNA transposon *piggyBac* (PB) by Institute of Developmental Biology and Molecular Medicine, Fudan University (Shanghai, China) as described previously [1]. The WT and RIIβ KO mice were maintained under specific pathogen-free conditions at 22 °C ± 2 °C and a 12 h light/dark cycle with free access to water and a normal chow-diet. Mice with similar body weight and fasting blood glucose levels were randomly assigned to experimental groups. Data acquisition and analysis were performed by investigators blinded to the experimental group. All animal handling protocols were approved by the Fudan University Animal Ethics Committees and followed the Chinese National Institute of Health guidelines on the care and use of animals.

2.2 Glucose tolerance test and hormone measurements

Mice were fasted overnight (14-16 h) before glucose administration. For glucose tolerance tests, mice received D-glucose at 2.0 g/kg BW either intraperitoneally (for IPGTT) or by oral gavage (for OGTT). Liraglutide was administered 30 min before glucose challenge during an IPGTT. Blood was drawn from the tail vein at 0, 15, 30, 60, and 90 min after glucose administration. Blood glucose levels were monitored using a glucometer. For hormone measurements, a 100 μl volume of blood was collected from the orbital sinus of fasted mice under isoflurane anaesthesia. The plasma was subsequently separated and used for hormone measurements. Insulin, glucagon and somatostatin were assayed using Ultra-Sensitive Mouse Insulin ELISA kit, Mouse Glucagon ELISA kit and Mouse Somatostatin ELISA kit, respectively.

2.3 Mouse islet isolation and INS-1(832/13) cell culture

Pancreatic islets were isolated from mice using collagenase digestion and density

gradient centrifugation as previously described [2]. Briefly, the pancreas was perfused and digested with collagenase XI solution at 37 °C for 16 min. Islets were then purified by centrifugation through a Ficoll step gradient. After overnight recovery, islets were hand-picked and used for subsequent analyses. Islets were infected with recombinant virus ADV-Syncollin-EGFP to label the insulin granules. The labelled islets were dispersed and then imaged using Olympus Spinning Disk confocal microscope.

INS-1(832/13) cells were maintained in RPMI 1640 containing 11.1 mmol/l glucose, 10% FBS, and 50 μmol/l 2-mercaptoethanol under standard conditions. Twenty-four hours after infection with recombinant virus ADV-Syncollin-EGFP, cells were transfected with siRNA targeting RIIβ using Lipofectamine RNAiMAX, as previously described [3]. Forty-eight hours after transfection, the cells were analysed by flow cytometry using CytoFLEX (Beckman).

2.4 Insulin secretion assay in mice and in isolated islets

For in vivo studies, blood was drawn from the tail vein of mice at 0 and 10 min after glucose administration. Plasma was separated and used for insulin measurement.

For in vitro studies, insulin release was measured using both static batch incubations and dynamic perifusion systems. In the static incubation analysis, islets were pre-incubated for 30 min in a Krebs–Ringer bicarbonate buffer (KRB) containing 2.8 mmol/l glucose and 0.1% bovine serum albumin. Groups of 7 islets were incubated for another 1 h in 2.8 mmol/l KRB, followed by 1 h stimulation with 16.7 mmol/l glucose and tested compounds. Supernatants were collected for insulin quantification.

For dynamic secretion profiling, the pre-treated 30 islets were loaded into a perifusion chamber. After 30 min equilibration with 2.8 mmol/l glucose KRB, islets were perifused at 0.15 ml/min with test solutions, with fractions collected at 1 min intervals for insulin analysis. Insulin was assayed using Ultra-Sensitive Mouse Insulin ELISA kit.

2.5 Immunoblotting and quantitative PCR analysis

Western blotting analysis was performed as described previously [4]. Briefly, total

protein extraction was performed by using RIPA buffer supplemented with a protease inhibitor cocktail. Ten micrograms of protein were separated by SDS-PAGE and electrically transferred to a polyvinylidene difluoride (PVDF) membrane (Millipore). After blocking, the blots were incubated with primary antibodies at 4 °C overnight. Signals were visualised using an ECL™ Western Blotting Detection system (GE Healthcare).

Total RNA was isolated with Total RNA Kit and converted into cDNA using One-step RT-gDNA-Digestion-SuperMix. Quantitative real-time PCR (qPCR) was conducted using Advanced qPCR-SYBR-MasterMix. Beta-actin was used as an internal control for normalisation.

RNA was extracted from isolated pancreatic islets and subjected to sequencing using Illumina BGIseq500 platform. Clean data was aligned to the reference gene set using Bowtie2 (v2.3.4.3). Gene expression quantification was performed using RSEM (v1.3.1). Differentially expressed genes were detected with criteria set as Q value $\leq$ 0.05 or FDR $\leq$ 0.001. KEGG enrichment analysis was performed on differential genes using the hypergeometric test implemented in Phyper, with a threshold of Q value $\leq$ 0.05.

The antibodies and oligonucleotide primers are listed in ESM Tables 1,2.

2.6 Electron microscopy

Pancreatic islets were equilibrated in KRB buffer containing 2.8 mmol/l glucose for 2 h and then treated with 16.7 mmol/l glucose for 10 min to induce secretory activity. Immediately after stimulation, islets were pre-fixed in 2.5% glutaraldehyde overnight at 4 °C. Samples were embedded in SPI-PON 812 resin. Ultrathin sections (70-90 nm) were cut using a diamond knife and mounted on copper grids. Images were captured using a transmission electron microscope (Tecnai G2 Spirit TWIN, FEI).

2.7 Electrophysiology

Whole-cell patch-clamp technique was employed to record K_{ATP} and Ca^{2+} currents

in single islet beta cells. K_{ATP} currents were recorded using perforated-patch recordings under both voltage step and voltage ramp protocols [5, 6]. For a voltage-ramp protocol, the recordings were established with glass pipettes (3-6 MΩ) held at -70 mV. The K_{ATP} currents were evoked with a ramp protocol from -120 mV to +40 mV over 4 s. Representative K_{ATP} current was measured between -80 mV and -60 mV. The current density was calculated as the measured current divided by the membrane capacitance (Cm). For a voltage step protocol, the K_{ATP} currents were evoked by a 10 mV incremental steps ranging from -100 mV to 0 mV (500 ms duration, 700 ms interval). K_{ATP} currents were quantified within the -80 mV to -60 mV, because K_{ATP} currents are prominent and minimally contaminated by other voltage-gated currents in this range [7]. The extracellular solution contained (in mmol/l): 129 NaCl, 5 $NaHCO_3$, 4.8 KCl, 1.2 KH_2PO_4, 1.2 $MgCl_2$, 2.5 $CaCl_2$, 10 HEPES, 2.8 Glucose, pH 7.2 adjusted with KOH. The ATP-free pipette solution contained (in mmol/l): 130 K-gluconate, 10 KCl, 1.5 $MgCl_2$, 10 HEPES, 1 EGTA, pH 7.2 adjusted with KOH. Perforated-patch configuration was achieved by adding pore-forming antibiotic amphotericin B (0.15 mg/mL) to the pipette solution. K_{ATP} currents were pharmacologically validated using Tolbutamide (TolB, 0.1 mmol/l) as an inhibitor.

For the recordings of the whole-cell Ca^{2+} currents, the extracellular solution contained (in mmol/l): 100 NaCl, 25 TEA-Cl, 3.5 KCl, 2 $MgCl_2$, 2 $CaCl_2$, 10 HEPES, 10 Glucose, pH 7.2 adjusted with KOH. To isolate Ca^{2+} currents, 2 μmol/l tetrodotoxin (TTX) and 1 mmol/l 4-aminopyridine (4-AP) were added to block voltage-gated Na^+ and K^+ channels, respectively. The pipette solution contained (in mmol/l): 115 $CsMeSO_3$, 25 TEA-Cl, 1 EGTA, 4 Mg-ATP, 0.5 Na-GTP, 10 HEPES, pH 7.2 adjusted with CsOH. Ca^{2+} currents were elicited using 200 ms voltage steps from -80 mV to +80 mV in 10 mV increments and analysed as previously described [8].

The voltage dependence of the Na^+ current inactivation was used to identify beta cells [9]. All electrophysiological recordings were performed at 30 °C using a feedback in-line heater (TC-324B; Warner Instruments). Data were filtered at 5 kHz and sampled at 10 kHz using an IPA Integrated Patch Amplifier (Sutter, USA) with Igor Pro (Wave

Metrics, Inc., USA) acquisition software.

2.8 Statistical analysis

The results are expressed as mean ± SEM. For two-group data sets analysis was done by unpaired two-tailed Student's *t* test. For three or more independent groups of data sets one-way ANOVA followed with Tukey corrected multiple comparison analysis was used. A p value <0.05 was considered statistically significant. Statistical analysis was performed using GraphPad Prism 8.2.1.

ESM Table 1. The oligonucleotide primers in this study.

	Accession	Forward	Reverse	Product size (bp)
mouse Cacna2d1	NM_001110843.1	GTGCAATGTAAATG TCGAACGG	TCAGGCAAAAGCATGA GTCTGT	86
mouse Cacnb2	NM_023116.5	CCCATCCGATTCAG ATGTGTCTT	TCTTTGCTTTTTCCAAC TGTGCC	95
mouse Cacnb3	NM_007581.4	GGGGAAAGTCACA GATGAAGCACCT	GGGTGGGCACTGAACC AGCTTAT	71
mouse Cacna1a	NM_001252059.1	GAAGTGAGTCCCCT GTCTGC	ACACCGACTTGGTAGG CTTCT	82
mouse Cacna1b	NM_007579.4	GCTTCTATTCCTGTG ACCGCTTT	CGATGTGGGGTGGCTA CTG	77
mouse Cacna1c	NM_009781.4	TCGTTTCAATGTTCC TAATGGGT	GAGGCTTATCCCGACA GCA	106
mouse Cacna1d	NM_001083616.2	TGTTAAGCGATGCC AGTTTAC	TGCGGTACTCTTCCAGT TTAC	91
mouse Cacna1e	NM_009782.3	TCACAAGTCTGACA CTCACCGAT	GAGAGAGGAGGTGCTT TCGTTC	74
mouse Cacna1g	NM_001112813	TGGGAGAACACTCG GAACTA	CAGTGACATCGGTGTA GCAG	77
mouse Calb1	NM_009788.4	GGCAACCATAGGAC TGTCA	TCGTTTCAGAATAAAGC CATAAT	120
mouse Calb2	NM_007586.2	TGATAGACAGTTGT GCCTGCGTT	GAAGCCTAAATCATACA GCGAAGGA	112
mouse Pvalb	NM_001330686.1	GGCAGTGGGGGTAT AGTAGAA	ATGAGCACTCAGTACC AAGCA	85
mouse S100g	NM_009789.2	GGCGATGGAGAAGT TAGTTACGAAG	GAGAGCGTGCGTTCAA TCAGT	114
mouse Actb	NM_007393.5	AACACCCCAGCCAT GTACGTAG	GAACCGCTCATTGCCG ATAGT	384

ESM Table 2. List of reagents used in this study.

REAGENT or RESOURCE	SOURCE	IDENTIFIER
Antibodies		
Mouse anti-RIIβ	BD transduction laboratory	615625
Rabbit anti-RI-α	Cell Signaling Technology	5675
Rabbit anti-Cα	Proteintech	27398-1
Rabbit anti-phospho-PKA Substrate	Cell Signaling Technology	9624
Rabbit anti-phospho-CREB (Ser133)	Cell Signaling Technology	9198
Mouse anti-HSP90	Proteintech	60318-1
Mouse anti-β actin	Proteintech	66009-1
HRP-conjugated goat anti-mouse IgG(H+L)	Proteintech	SA00001-1
HRP-conjugated goat anti-rabbit IgG (H+L)	Jackson ImmunoResearch	111-035-003
Chemicals, peptides, and kits		
Liraglutide	Novo Nordisk	N/A
Glucagon	Novo Nordisk	N/A
Follicle-Stimulating Hormone	Merck	N/A
Forskolin	MedChemExpress	HY-15371
Tolbutamide	MedChemExpress	HY-B0401
Diazoxide	MedChemExpress	HY-B1140
Collagenase XI	Sigma	C7657
Histopaque-1077	Sigma	10771
Histopaque-1119	Sigma	11191
RPMI 1640 Medium	Thermo Fisher Scientific	31800022
FBS	Gibco	A5669701
Trypsin	Gibco	15090046
Protease inhibitor cocktail	Roche	04693159001
One-step RT-gDNA-Digestion-SuperMix	Yeasen Biotech (Shanghai China)	1151ES10
Advanced qPCR-SYBR-MasterMix	Yeasen Biotech (Shanghai China)	11185ES08
SPI-PON 812 resin	SPI Supplies	02659-AB
Mouse Insulin ELISA Kit	Crystalchem	90080
Mouse GC (Glucagon) ELISA Kit	Elabscience	E-EL-M0555
Mouse SST (Somatostatin) ELISA Kit	Elabscience	E-EL-M1086

Deposited Data		
Raw data files and processed data files for RNA-seq	This study	SRA: SRX29481113
Experimental models: Cell lines		
INS-1(832/13) cells	Gift from Tianru Jin (University of Toronto)	N/A
Virus strains		
ADV-Syncollin-EGFP	Ruimian Biotech	N/A
Software and algorithms		
ImageJ 2/Fiji	NIH	https://imagej.net/ij/
Leica Application Suite X (LAS X) 3.7.4.23163	Leica Microsystems	http://leica-microsystems.com
Graphpad 8.2.1	GraphPad Software Inc	https://www.graphpad.com/
Cellpose 3	HHMI	https://www.cellpose.org/
Bowtie2 2.3.4.3	Johns Hopkins University	http://bowtie-bio.sourceforge.net/bowtie2
RSEM 1.3.1	N/A	https://github.com/deweylab/RSEM
Igor Pro acquisition software 9.0.1.2	WaveMetrics	https://www.wavemetrics.com/
Other		
Illumina BGIseq500 platform	BGI-Shenzhen	N/A
Feedback in-line heater	Warner Instruments	TC-324B
IPA Integrated Patch Amplifier	Sutter Instrument	N/A
Tecnai G2 Spirit TWIN	FEI	N/A
Diamond Knife	Diatome	N/A

ESM Figures

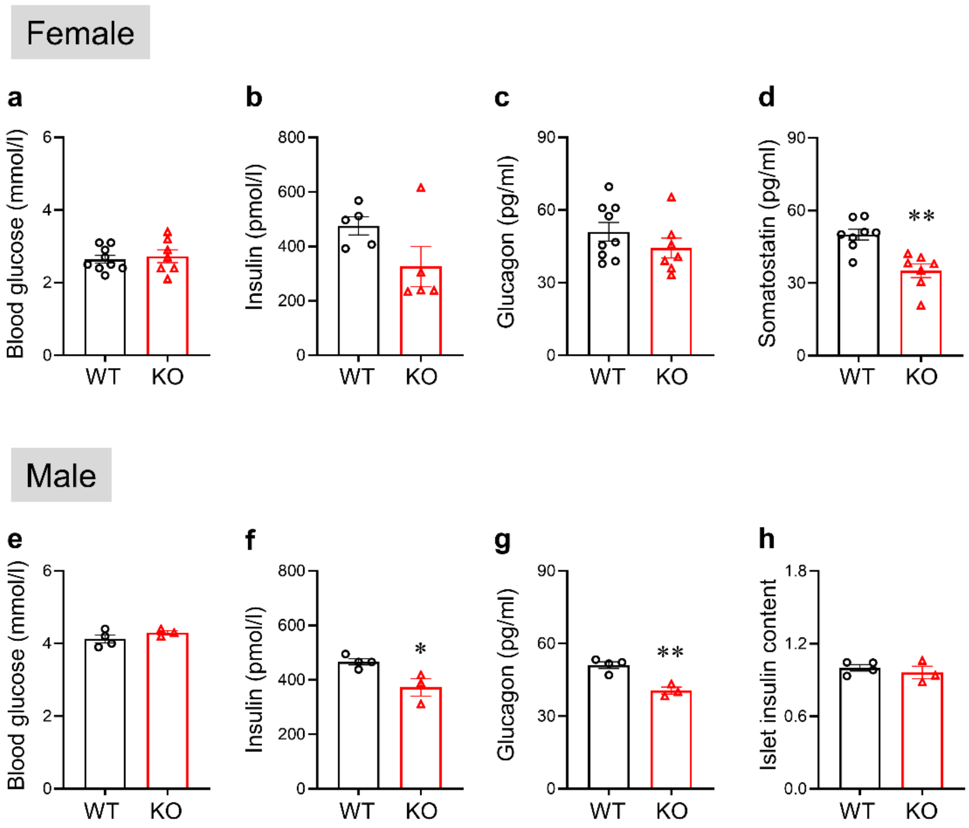

ESM Figure 1. RIIβ KO mice had altered serum hormone levels and comparable insulin content in islets, compared to WT mice. Female (a-d) and male (e-h) mice were fasted overnight. The RIIβ KO mice with comparable fasting blood glucose level with WT mice were used for blood sampling. The serum levels of insulin (b, f), glucagon (c, g) and somatostatin (d) were determined using ELISA kits. n=5-9 WT, n=5-7 RIIβ KO (a-d); n=4 WT, n=3 RIIβ KO (e-g). (h) A total of 30 islets were assessed for islet insulin content. n=4 WT, n=3 RIIβ KO. Data are mean ± SEM; **p*<0.05, ***p*<0.01; unpaired *t* test

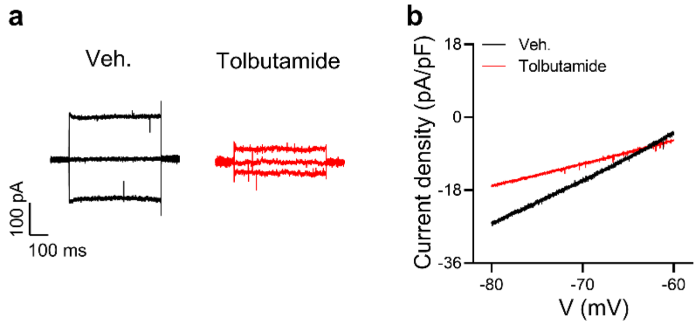

ESM Figure 2. Whole-cell K_{ATP} currents were validated by exposing WT beta cells to a K_{ATP} channel inhibitor. Whole-cell currents were recorded from beta cells of isolated islets before and after Tolbutamide (0.1 mmol/l) treatment. (a) Representative traces of K_{ATP} currents from -80 mV to -60 mV using voltage step protocol. (b) Representative traces of K_{ATP} currents from -80 mV to -60 mV using voltage ramp protocol. Veh., vehicle (extracellular solution for K_{ATP} current recording)

References

1. Ding S, Wu X, Li G, Han M, Zhuang Y, Xu T (2005) Efficient transposition of the piggyBac (PB) transposon in mammalian cells and mice. Cell 122(3): 473–483. https://doi.org/10.1016/j.cell.2005.07.013
2. Jiang Y, Wu D, Ni Y, et al. (2025) Peptide hormone spexin restores beta cell function and improves glycaemic control in mice via regulation of the sodium-potassium pump. Diabetologia. https://doi.org/10.1007/s00125-025-06596-z
3. Jiang Y, Zhu L, Wu D, et al. (2022) Type IIB PKA is highly expressed in beta cells and controls cell proliferation via regulating Cyclin D1 expression. FEBS J 289(10): 2865–2876. https://doi.org/10.1111/febs.16302
4. Jiang Y, Wang Z, Ma B, et al. (2018) GLP-1 Improves Adipocyte Insulin Sensitivity Following Induction of Endoplasmic Reticulum Stress. Front Pharmacol 9: 1168. https://doi.org/10.3389/fphar.2018.01168
5. Tarasov A, Dusonchet J, Ashcroft F (2004) Metabolic regulation of the pancreatic beta-cell ATP-sensitive K+ channel: a pas de deux. Diabetes 53 Suppl 3: S113–122. https://doi.org/10.2337/diabetes.53.suppl_3.s113
6. Williams BA, Smith PA, Leow K, Shimizu S, Gray DW, Ashcroft FM (1993) Two types of potassium channel regulated by ATP in pancreatic B cells isolated from a type-2 diabetic human. Pflugers Arch 423(3-4): 265–273. https://doi.org/10.1007/bf00374405
7. Oduori OS, Murao N, Shimomura K, et al. (2020) Gs/Gq signaling switch in β cells defines incretin effectiveness in diabetes. J Clin Invest 130(12): 6639–6655. https://doi.org/10.1172/jci140046
8. Schulla V, Renstrom E, Feil R, et al. (2003) Impaired insulin secretion and glucose tolerance in beta cell-selective Ca(v)1.2 Ca2+ channel null mice. EMBO J 22(15): 3844–3854. https://doi.org/10.1093/emboj/cdg389
9. Göpel S, Kanno T, Barg S, Galvanovskis J, Rorsman P (1999) Voltage-gated and resting membrane currents recorded from B-cells in intact mouse pancreatic islets. J Physiol 521 Pt 3(Pt 3): 717–728. https://doi.org/10.1111/j.1469-7793.1999.00717.x
